# Genome-wide RNA-Sequencing analysis identifies a distinct fibrosis gene signature in the conjunctiva after glaucoma surgery

**DOI:** 10.1038/s41598-017-05780-5

**Published:** 2017-07-17

**Authors:** Cynthia Yu-Wai-Man, Nicholas Owen, Jonathan Lees, Aristides D. Tagalakis, Stephen L. Hart, Andrew R. Webster, Christine A. Orengo, Peng T. Khaw

**Affiliations:** 1 National Institute for Health Research (NIHR) Biomedical Research Centre at Moorfields Eye Hospital NHS Foundation Trust and UCL Institute of Ophthalmology, London, EC1V 9EL United Kingdom; 2 UCL Institute of Ophthalmology, London, EC1V 9EL United Kingdom; 3 Bioinformatics Research Group, UCL Institute of Structural and Molecular Biology, London, WC1E 6BT United Kingdom; 4 Experimental and Personalised Medicine Section, UCL Great Ormond Street Institute of Child Health, London, WC1N 1EH United Kingdom

## Abstract

Fibrosis-related events play a part in most blinding diseases worldwide. However, little is known about the mechanisms driving this complex multifactorial disease. Here we have carried out the first genome-wide RNA-Sequencing study in human conjunctival fibrosis. We isolated 10 primary fibrotic and 7 non-fibrotic conjunctival fibroblast cell lines from patients with and without previous glaucoma surgery, respectively. The patients were matched for ethnicity and age. We identified 246 genes that were differentially expressed by over two-fold and *p* < 0.05, of which 46 genes were upregulated and 200 genes were downregulated in the fibrotic cell lines compared to the non-fibrotic cell lines. We also carried out detailed gene ontology, KEGG, disease association, pathway commons, WikiPathways and protein network analyses, and identified distinct pathways linked to smooth muscle contraction, inflammatory cytokines, immune mediators, extracellular matrix proteins and oncogene expression. We further validated 11 genes that were highly upregulated or downregulated using real-time quantitative PCR and found a strong correlation between the RNA-Seq and qPCR results. Our study demonstrates that there is a distinct fibrosis gene signature in the conjunctiva after glaucoma surgery and provides new insights into the mechanistic pathways driving the complex fibrotic process in the eye and other tissues.

## Introduction

Glaucoma is the leading cause of irreversible blindness worldwide and fibrosis is the critical determinant of the long-term surgical success after glaucoma surgery. Histopathologic findings of failed fibrotic capsules have indicated a key role of the fibroblast and the production of extracellular matrix components in the pathophysiology of fibrotic encapsulation in glaucoma surgery^1^. However, fibrosis is a complex multifactorial disease and little is known about the diverse molecular mechanisms and pathways underlying the fibrotic response. Identifying the dysregulated genes will be important in understanding the complex network of signalling pathways driving the fibrotic process and in finding potential novel therapeutic targets and biomarkers of disease severity and prognosis in fibrotic eye diseases.

It is well established that certain groups of patients, for example Afro-Caribbean people, scar worse than others^2, 3^. However, reliable biomarkers to stratify the risk of scarring and post-surgical fibrosis in the eye and other tissues are currently missing. Being able to predict a patient’s risk of scarring according to their genetic profile holds great potential to the development of a more personalised and stratified therapy in ocular fibrosis^4^. A major hurdle has been the lack of availability of human tissues for research and the small surgical specimen sizes yielding limited amounts and poor quality RNA. RNA-Sequencing (RNA-Seq) is a powerful and highly sensitive technology that allows the whole transcriptome to be studied compared to DNA microarrays and also requires smaller amounts of RNA^5, 6^.

In this study, we have carried out a genome-wide RNA-Seq analysis to compare the gene expression profiles of fibrotic and non-fibrotic human conjunctival fibroblast cell lines between patients with and without previous glaucoma surgery, respectively. We have also mapped the enrichment of differentially expressed genes to specific gene ontology and protein networks, and identified distinct changes in signalling pathways that could contribute to the fibrotic process and clinical phenotype.

## Results

### Patient Demographics

We performed RNA-Sequencing on 10 fibrotic fibroblast (FF3, FF10, FF13, FF14, FF15, FF16, FF17, FF18, FF20, FF21) and 7 non-fibrotic fibroblast (NF1, NF4, NF7, NF8, NF9, NF10, NF11) cell lines. The patients were matched for ethnicity and age. The majority of patients in both groups were white Caucasians: 7 white Caucasians (70%), 1 Asian, and 2 Afro-Caribbeans in the FF group; 6 white Caucasians (86%) and 1 Asian in the NF group (Table 1). There were also no statistically significant differences in age between the two groups with a mean age of 41.7 ± 17.1 years for FF patients and 52.0 ± 26.5 years for NF patients (*p* = 0.344).

**Table 1:** Patient demographics for the FF and NF groups.

	Fibrotic (FF)	Non-Fibrotic (NF)
Number	10	7
Age, mean in years ± SD	41.7 ± 17.1	52.0 ± 26.5
Gender (M/F)	5 M/5 F	6 M/1 F
Ethnicity	7 Caucasians	6 Caucasians
	1 Asian	1 Asian
	2 Afro-Caribbeans	
Type of glaucoma	7 POAG	3 POAG
	3 congenital	3 secondary
		1 congenital
Pre-operative intraocular pressures, mean in mmHg ± SD	19.4 ± 13.0	27.0 ± 7.9
Best-corrected visual acuity, mean in logMAR (range)	0.7 (0 to 1)	0.3 (0 to 0.6)
Cup-disc ratio, mean (range)	0.9 (0.7 to 1.0)	0.8 (0.8 to 0.9)
Anti-glaucoma eye drops, mean (range)	3.2 (0 to 5)	3.9 (3 to 5)
Previous glaucoma surgeries, mean (range)	1.7 (1 to 3)	0

The FF patients had marked conjunctival fibrosis from previous glaucoma surgeries (mean = 1.7, range = 1 to 3). The mean central bleb area, maximal bleb area, bleb height, and bleb vascularity were 1.8 ± 1.0, 1.8 ± 1.0, 1.7 ± 0.8, and 3.7 ± 1.0, respectively. The FF group had worse best-corrected visual acuity with a mean logMAR vision of 0.7 compared to 0.3 for the NF group (*p* = 0.028). The FF patients also had a mean pre-operative intraocular pressure (IOP) of 19.4 ± 13.0 mmHg while the NF patients had a mean pre-operative IOP of 27.0 ± 7.9 mmHg (*p* = 0.191). All patients had advanced optic disc cupping with a mean cup-disc ratio of 0.9 and 0.8 in the FF and NF groups, respectively (*p* = 0.313).

### RNA-Seq analysis identifies a distinct fibrosis gene signature in the conjunctiva

**Figure 1:**
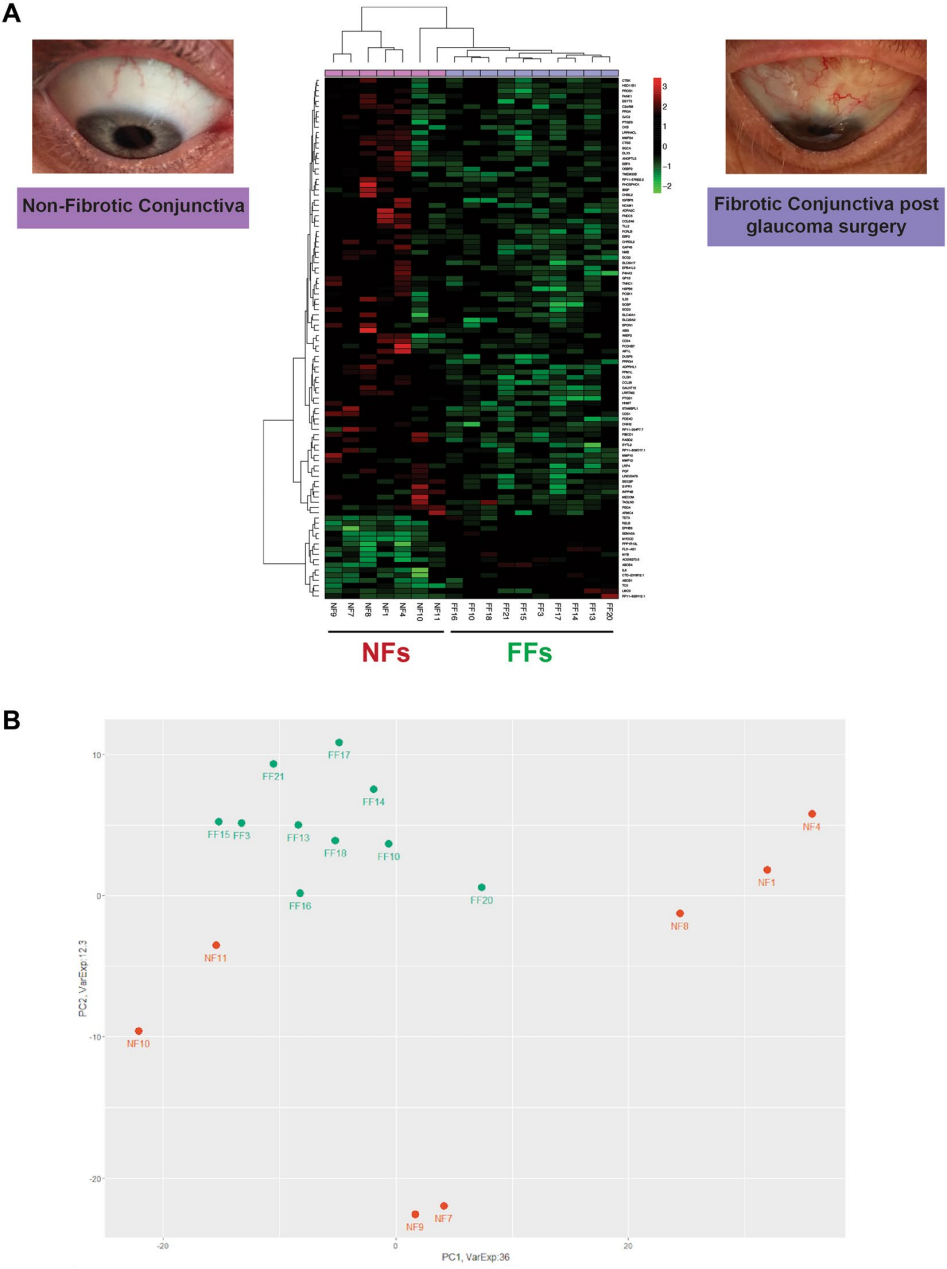
(**A**) Heat map of differentially expressed genes between FFs and NFs. The 100 genes shown in the heat map were selected as being the most significant changes, i.e. sorted by *p* values with the smallest and most significant at the top. The genes were clustered using hierarchical average linkage clustering and euclidean distances using the R package for Nonnegative Matrix Factorization (NMF)^75^. (**B**) Principal component analysis (PCA) was performed using DESeq2 on the regularised log transformed count data.

We extracted high quality RNA from all the fibroblast cell lines (RNA integrity number equivalent ≥9.8). Table 2 shows the total reads sequenced, the intragenic/exonic/intronic/intergenic rates, the number of genes detected, and the mean coverage in each sample. We identified a distinct fibrosis gene expression profile in the conjunctiva after glaucoma surgery (Fig. 1A). A total of 246 genes were differentially expressed in fibrotic fibroblast (FFs) cell lines compared to non-fibrotic fibroblast (NFs) cell lines with more than a two-fold change and which were statistically significant (*p* < 0.05; Table S1). Out of the 246 genes, 46 genes were upregulated and 200 genes were downregulated in FFs compared to NFs. The reason for the significantly larger number of downregulated compared to upregulated genes might be because many genes encoding the large family of extracellular matrix proteins, growth factors, signalling molecules, and tumour suppressor genes were significantly downregulated in FFs compared to NFs.

**Table 2:** The total reads sequenced, the intragenic/exonic/intronic/intergenic rates, and the number of genes detected in each sample are presented here.

Sample	Total reads sequenced	Mapped	Mapped unique	Intragenic rate	Exonic rate	Intronic rate	Intergenic rate	Genes detected	Mean coverage-High	Mean coverage-Medium	Mean coverage-Low
FF3	42023726	37592844	27995882	0.948	0.884	0.065	0.052	15674	376.99	16.37	3.82
FF10	37112186	30779958	22735702	0.926	0.862	0.065	0.073	15421	371.14	12.91	3.24
FF13	18788002	16758670	13529630	0.945	0.884	0.061	0.054	14611	212.97	7.47	1.73
FF14	59651682	54662138	36210246	0.947	0.885	0.062	0.052	16031	532.51	22.87	5.98
FF15	34965998	32923072	24999524	0.948	0.886	0.062	0.052	15355	366.59	14.58	3.41
FF16	45049566	41338508	28216626	0.944	0.854	0.089	0.056	15742	395.65	15.88	3.93
FF17	42367044	38687824	26996622	0.930	0.865	0.066	0.070	15508	408.80	16.06	4.01
FF18	38247446	35096216	22061352	0.932	0.873	0.059	0.068	15612	340.66	12.77	3.01
FF20	45156828	41265828	28362946	0.938	0.872	0.066	0.062	16034	399.06	16.36	3.98
FF21	62590502	54318504	35711252	0.943	0.877	0.065	0.057	16030	506.28	21.60	5.31
NF1	46016224	40107626	28253026	0.926	0.862	0.064	0.074	16032	395.65	16.10	4.07
NF4	39105994	36621378	27604732	0.929	0.871	0.059	0.070	15517	386.04	14.99	3.64
NF7	51543864	47002456	33462460	0.937	0.875	0.062	0.063	16027	444.52	19.48	4.56
NF8	39174808	35558266	26244832	0.925	0.864	0.061	0.075	15803	381.37	14.67	3.54
NF9	44996604	40845860	29106082	0.919	0.855	0.063	0.081	15878	383.28	16.38	3.86
NF10	51037730	45634882	32393242	0.933	0.867	0.066	0.067	15735	466.67	18.78	4.39
NF11	46797824	43182836	31797826	0.938	0.874	0.063	0.062	15951	426.46	18.68	4.24

High, medium, and low mean coverage calculations are based on the top 1000, middle 1000, and bottom 1000 expressed transcripts, respectively.

**Figure 2:**
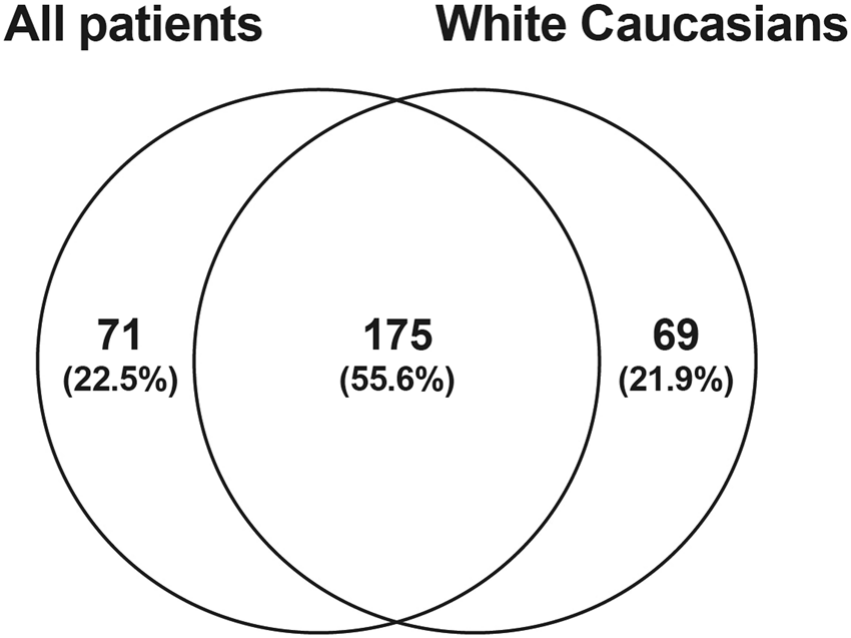
Venn diagram listing shared and unique genes in the ‘all patients’ and ‘white Caucasians’ groups. Percentages of patients are shown in brackets.

Principal component analysis revealed tight clustering of FFs whereas there was more variability in NFs (Fig. 1B). As ethnicity can affect the wound healing response after glaucoma surgery, we also performed an additional Caucasian FF-Caucasian NF comparison (Fig. 2). The majority of patients in our cohort were white Caucasians and we found a large overlap (175 out of the 246 genes) between the ‘all patients’ and ‘white Caucasians’ group comparisons (Table S2).

### Gene Ontology Analysis

**Figure 3:**
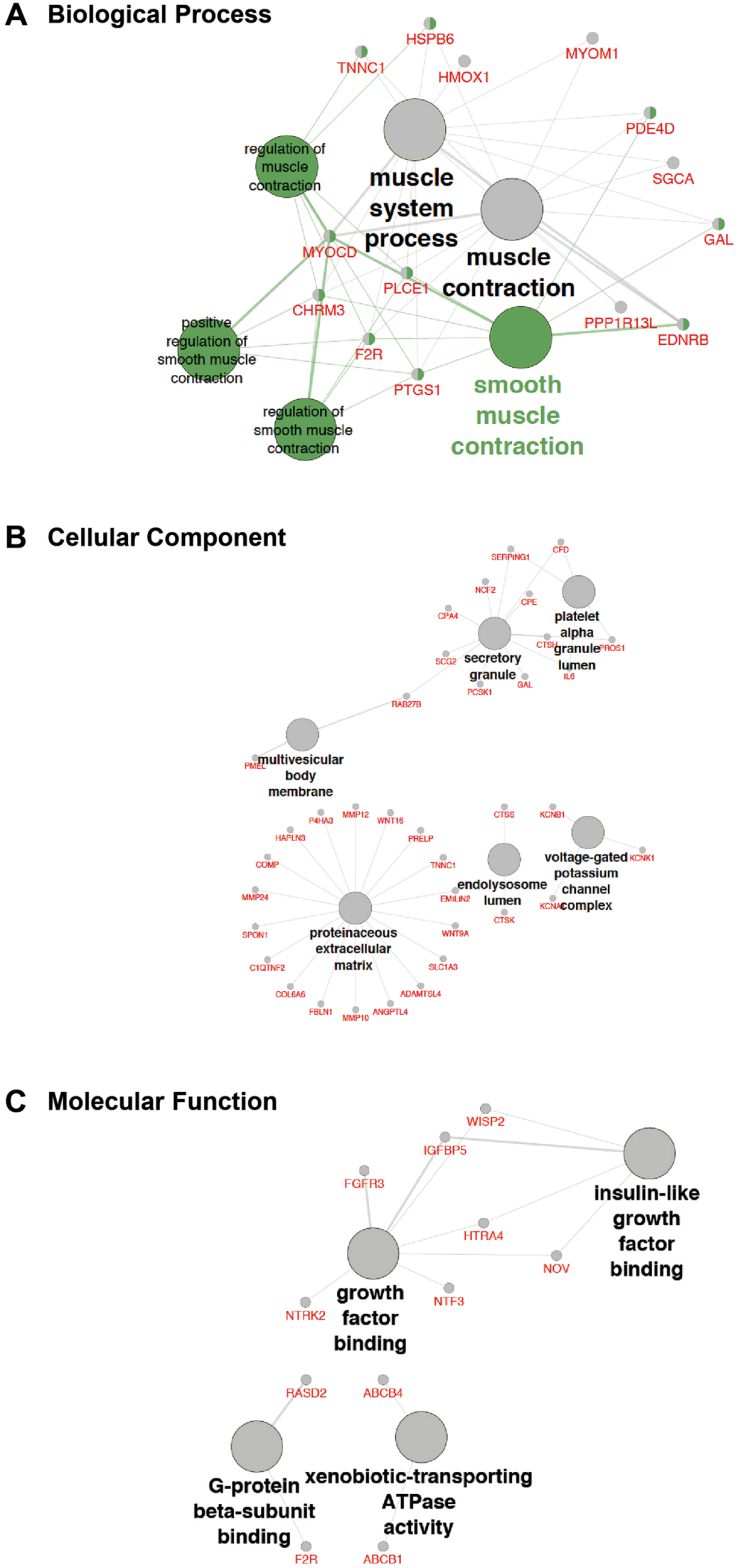
Enriched gene ontology groups: (**A**) Biological process, (**B**) Cellular component, (**C**) Molecular function. The differentially expressed genes list was analysed using ClueGo in Cytoscape. Gene node shading indicates shared associations with each term.

We next carried out detailed GO (gene ontology) enrichment analysis of the 246 differentially expressed genes (Table S4). Figure S3 shows the directed acyclic graph (DAG) view of the GO analysis. Enriched ontology groups shown in red included regulation of smooth muscle contraction, proteinaceous extracellular matrix, regulation of secretion, the mitogen-activated protein kinase (MAPK) cascade, and angiogenesis. For the biological process, smooth muscle contraction and muscle contraction were enriched ontology groups (Fig. 3A). The *MYOCD* gene encodes myocardin, a smooth muscle-specific transcriptional co-activator of serum response factor (SRF), and its expression was significantly upregulated in FFs compared to NFs (Table S1). The *CHRM3* gene encodes a muscarinic acetylcholine receptor M_3_ that causes smooth muscle contraction and its expression was also significantly increased in FFs compared to NFs.

For the cellular component, proteinaceous extracellular matrix and secretory granule were enriched ontology groups (Fig. 3B). Among the proteinaceous extracellular matrix, the *COL6A6* gene and the *P4HA3* gene that encodes prolyl 4-hydroxylase, a key enzyme in collagen synthesis, were significantly downregulated in FFs compared to NFs. The *MMP-10, MMP-12* and *MMP-24* genes encode matrix metalloproteinases and their expression were also significantly decreased in FFs compared to NFs. The *FBLN1* gene encodes the extracellular matrix component, fibulin-1, and was significantly downregulated in FFs compared to NFs. For the regulation of secretion, the *IL-6* gene was significantly upregulated whereas the *IL-33* and *CD34* genes were downregulated in FFs compared to NFs (Table S4).

For the molecular function, growth factor binding and insulin-like growth factor binding were enriched ontology groups (Fig. 3C). The *FGFR3* gene encodes the fibroblast growth factor receptor 3 and was significantly downregulated in FFs compared to NFs. The *IGFBP5* gene encodes insulin-like growth factor-binding protein 5 and its expression was also significantly decreased in FFs compared to NFs. The *WISP2 (CCN5)* gene is a new transcriptional regulator of the TGFβ signaling pathway and its expression was significantly downregulated in FFs compared to NFs. The *NOV (CCN3)* gene is a negative regulator of *CTGF (CCN2)* and its expression was also significantly decreased in FFs compared to NFs.

### KEGG, Disease association, Pathway commons, WikiPathways Analyses

We also performed detailed KEGG (Kyoto Encyclopedia of Genes and Genomes), disease association, pathway commons, and WikiPathways analyses of the 246 differentially expressed genes (Tables S5 to S8). There were many similarities between the GO analysis and the other enrichment analyses. In the KEGG pathway (ECM-receptor interaction and metabolic pathways), the *COL6A6*, *COMP* and *P4HA3* genes were significantly downregulated in FFs compared to NFs (Table S5). In the KEGG pathway (MAPK signalling pathway), the *RELB* gene was also significantly upregulated whereas the *FGFR3* and *DUSP5* genes were downregulated in FFs compared to NFs.

In the disease association analysis (inflammation), the *IL-6* gene expression was significantly increased whereas the *IL-33* gene expression was decreased in FFs compared to NFs (Table S6). In the disease association analysis (neoplasms, cancer or viral infections, breast neoplasms, neuroblastoma), the *LMO3*, *MYB* and *BIRC3* oncogenes were also significantly upregulated whereas the *WISP2, IGFBP5* and *RASSF2* tumour suppressor genes were significantly downregulated in FFs compared to NFs.

In addition, in the pathway commons analysis (IGF1 pathway, thrombin/protease-activated receptor pathway, signalling events), the *MYOCD* gene was significantly upregulated in FFs compared to NFs (Table S7). In the pathway commons analysis (IGF1 pathway, IFN-gamma pathway, IL3-mediated signalling events), the *IL-6*, *RELB* and *PPP1R13L* gene expression was also significantly increased whereas the *DUSP5* and *FGFR3* gene expression was decreased in FFs compared to NFs.

In the WikiPathways analysis (matrix metalloproteinases), the *MMP-10*, *MMP-12* and *MMP-24* genes were significantly downregulated in FFs compared to NFs (Table S8). In the WikiPathways analysis (myometrial relaxation and contraction pathways), the *IL-6* gene expression was also significantly increased whereas the *IGFBP5* gene expression was decreased in FFs compared to NFs.

### Protein Network Analysis

We further implemented a novel network analysis method utilising the STRING v10 network data to identify clusters of genes showing similar significant sets of gene associations and similar patterns of differential expression (Table S9). This strategy allows us to leverage the large amount of information held in the STRING database to further characterise the RNA-Seq data by looking for shared neighbourhoods in the STRING network that show similar differential expression. Since this network-based method does not use discrete groups (pathway groupings)‚ as applied to the standard enrichment approach, it can provide a complimentary and more global view on the gene expression changes.

**Figure 4:**
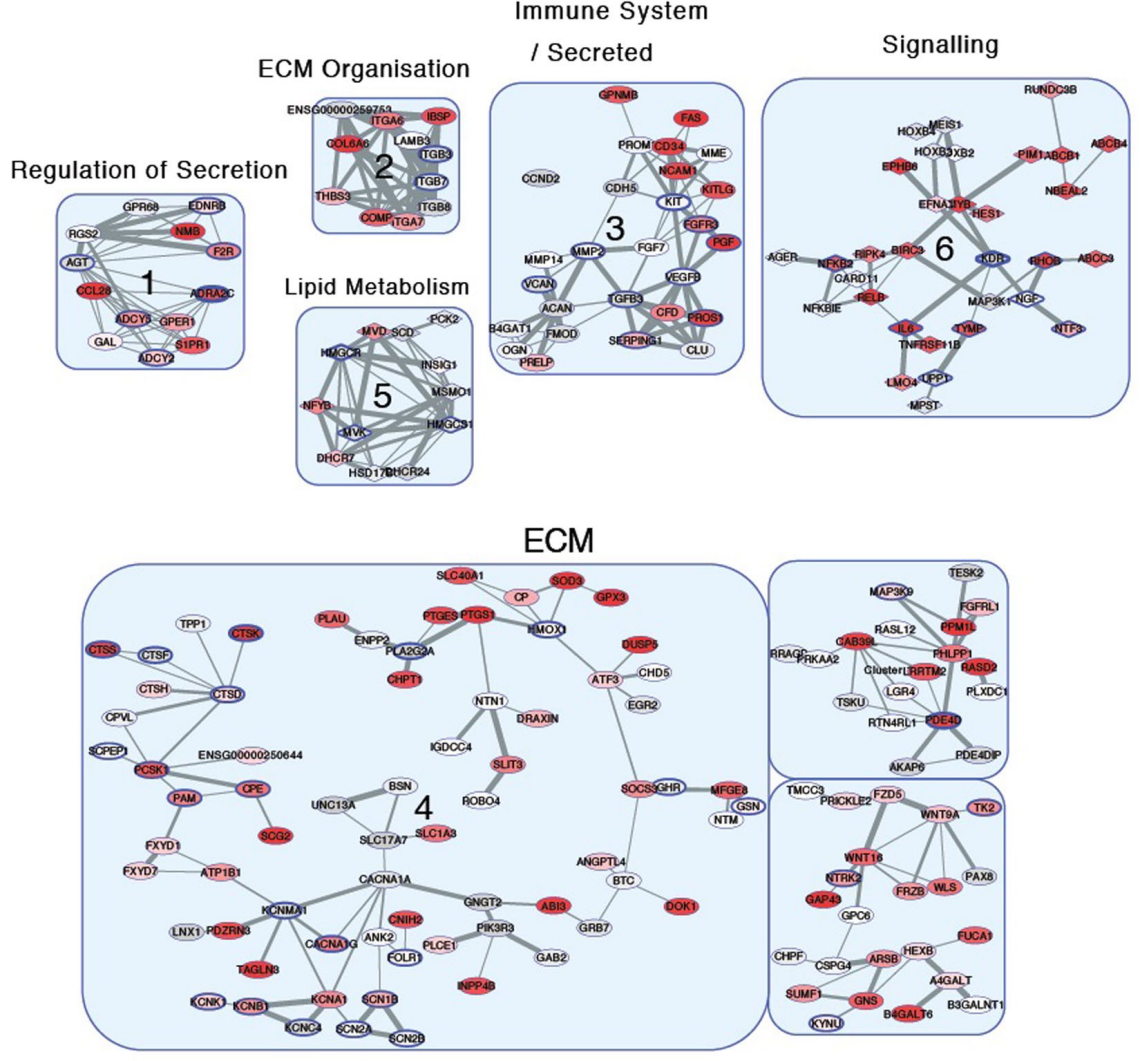
High-level modules were identified by a network clustering algorithm (see methods) using the STRING network data. Redness-fill of a node corresponds to the adjusted *p* value from the RNA-Seq analysis (redder nodes have lower *p* values). Diamonds correspond to upregulated genes whilst ellipses correspond to downregulated genes in FFs compared to NFs. The thickness of the node boundary corresponds to the number of drugs available to bind the protein. The GO term most strongly associated with the cluster is shown in the label. ECM = Extracellular matrix.

Like the GO analysis, extracellular organisation and components were also enriched in clusters 2 and 4 of the network clustering analysis (Fig. 4). The *COL6A6*, *COMP, PRG4, FBLN1* and *P4HA3* genes were significantly downregulated in FFs compared to NFs (Table S9). Several matrix metalloproteinases genes (*MMP-2, MMP-10, MMP-14, MMP-15, MMP-24*) were also downregulated in FFs compared to NFs. In addition, inflammatory cytokines and immune mediators were enriched in clusters 3 and 6 of the network clustering analysis. The *IL-6*, *RELB*, *PPP1R13L* and *NFKB2* genes were significantly upregulated in FFs compared to NFs (Table S9). Conversely, the *IL-33* and *CD34* gene expression were significantly decreased in FFs compared to NFs. In cluster 4, the *DUSP5* gene negatively regulates members of the MAPK family that are associated with cellular proliferation and differentiation and its expression was significantly decreased in FFs compared to NFs.

In cluster 6 of the network clustering analysis, the *MYOCD* and *CHRM3* genes were also significantly upregulated in FFs compared to NFs (Table S9). In clusters 3 and 4, the *FGFR3* and *IGFBP5* genes were downregulated in FFs compared to NFs. Moreover, we found several genes associated with cancer to be differentially expressed in FFs compared to NFs. In cluster 6, the *LMO3*, *MYB*, and *BIRC3* oncogenes were significantly upregulated in FFs compared to NFs. The *WISP2, IGFBP5* and *RASSF2* genes are tumour suppressor genes and were also significantly downregulated in FFs compared to NFs in cluster 4 of the network clustering analysis.

### Validation by Real-time quantitative PCR

We further validated 11 selected genes that were highly upregulated or downregulated in the RNA-Seq analysis using real-time qPCR (Table 3). For the upregulated genes, the *MYOCD, LMO3, IL-6* and *RELB* gene expression were significantly increased in the RNA-Seq and RT-qPCR results. For the downregulated genes, the *PRG4, CD34, IL-33, MMP-10, WISP2, COL6A6* and *IGFBP5* gene expression were significantly decreased in the RNA-Seq and RT-qPCR results.

**Table 3:** Validation of highly upregulated or downregulated genes using real-time quantitative PCR.

Gene	RT-qPCR		RNA-Seq	
	Fold change (FF vs NF)	*p* value	Fold change (FF vs NF)	*p* value
*MYOCD*	+30.22**	0.005	+11.45**	0.006
*LMO3*	+7.11*	0.046	+9.13**	0.003
*IL-6*	+2.70**	0.001	+3.04***	0.0003
*RELB*	+1.74*	0.017	+2.14**	0.001
*PRG4*	−47.62*	0.023	−49.61***	0.0006
*CD34*	−28.57*	0.033	−14.75**	0.003
*IL-33*	−27.78*	0.043	−19.90***	0.0005
*MMP-10*	−20.06*	0.018	−16.54***	0.0003
*WISP2*	−15.15*	0.027	−8.78**	0.009
*COL6A6*	−13.39*	0.033	−7.41***	0.0008
*IGFBP5*	−6.29*	0.022	−3.94**	0.002

All mRNA values were normalised relative to that of GAPDH and triplicate experiments were performed for each gene. Statistically significant differences were expressed as *p < 0.05; **p < 0.01; ***p < 0.001.

**Figure 5:**
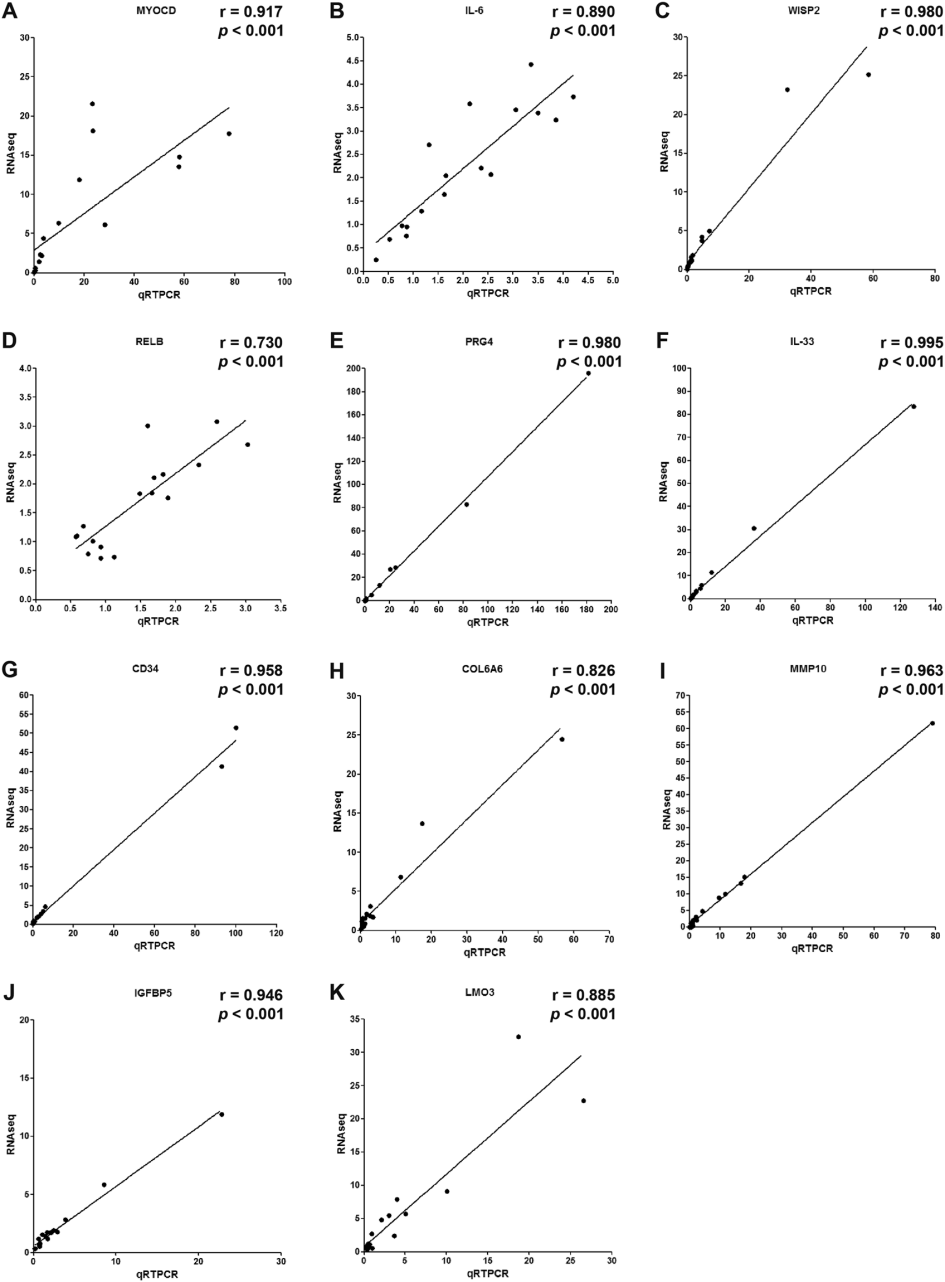
The Spearman’s correlation r and corresponding *p* values of the RNA-Seq and RT-qPCR results were performed using the mean values obtained from all samples normalised to either NFs or FFs: (**A**) *MYOCD*, (**B**) *IL-6*, (**C**) *WISP2*, (**D**) *RELB*, (**E**) *PRG4*, (**F**) *IL-33*, (**G**) *CD34*, (**H**) *COL6A6*, (**I**) *MMP-10*, (**J**) *IGFBP5*, (**K**) *LMO3* genes.

We also studied the correlation between the RNA-Seq and RT-qPCR analyses and found a strong correlation between the two sets of results. The Spearman correlation coefficient (r) was 0.917 (*p* < 0.001) for the *MYOCD* gene, 0.890 (*p* < 0.001) for the *IL-6* gene, 0.980 (*p* < 0.001) for the *WISP2* gene, 0.730 (*p* < 0.001) for the *RELB* gene, 0.980 (*p* < 0.001) for the *PRG4* gene, 0.995 (*p* < 0.001) for the *IL-33* gene, 0.958 (*p* < 0.001) for the *CD34* gene, 0.826 (*p* < 0.001) for the *COL6A6* gene, 0.963 (*p* < 0.001) for the *MMP-10* gene, 0.946 (*p* < 0.001) for the *IGFBP5* gene, and 0.885 (*p* < 0.001) for the *LMO3* gene (Fig. 5A to K).

## Discussion

Here we present the first genome-wide RNA-Sequencing study in human conjunctival fibrosis. We have studied the differentially expressed genes between fibrotic and non-fibrotic primary human conjunctival fibroblast cell lines from patients with and without previous glaucoma surgery, respectively. Previous microarray studies in the rabbit^7^ and rat^8^ eyes have reported 315 and 923 significantly altered genes after glaucoma filtration surgery, respectively. There has only been one human study of seven capsules of failed glaucoma valves using the RT² Profiler PCR Array, and the authors have found that 39 of the 84 tested genes had more than two-fold differential gene expression in three or more of the Tenon’s capsules^9^. Common genes between the three studies and our study include extracellular matrix components and matrix metalloproteinases.

We found that the *MYOCD* gene was significantly upregulated in FFs compared to NFs. Myocardin is a critical cofactor of serum response factor (SRF) in the transcriptional program regulating smooth muscle cell differentiation^10^, and can activate smooth muscle gene expression in a variety of non-muscle cell types via its association with SRF^11^. Myofibroblasts are contractile smooth muscle-like cells that control tissue repair and remodelling, and persistent myofibroblast activation is associated with pathological fibrosis and scarring^12–14^. Alpha smooth muscle actin (αSMA) is also a classical smooth muscle target gene of MYOCD and SRF^15, 16^, and αSMA expression upregulates fibroblast contractile activity^17^. SRF is a master regulator of cytoskeletal gene expression^18, 19^, and the Myocardin-related transcription factor/Serum response factor (MRTF/SRF) pathway has been linked to ocular^20–22^, vascular^23^, skin^24^, and lung fibrosis^25^.

Fetal skin wound healing is scarless and fundamentally different from adult wound healing^26–29^. Scarless fetal wound healing is characterised by decreased levels of potent inflammatory cytokines such as IL-6^30^, and the *IL-6* gene was significantly upregulated in FFs compared to NFs. Scarless fetal wound healing is also associated with high levels of hyaluronic acid^27, 28^ that increases the expression of proteoglycan 4 (PRG4)^31^. Similarly, our results show that the *PRG4* gene was significantly upregulated in NFs compared to FFs. IL-6 controls the effector characteristics of various T cell subsets including Th17 cells, Th22 cells^32^, and also plays an important role in trachomatous conjunctival fibrosis^33^, pulmonary fibrosis^34^, peritoneal fibrosis^35^, renal interstitial fibrosis^36^, and cancer-associated fibroblasts^37^. Conversely, the *IL-33* gene was significantly downregulated in FFs compared to NFs. IL-33 has a protective effect and reduces cardiac hypertrophy and fibrosis after experimental myocardial infarction through ST2 signalling^38^.

Furthermore, we found that the *RELB* and *PPP1R13L* genes were significantly upregulated in FFs compared to NFs. The *RELB* gene is part of the NFkB family, which is a master regulator of inflammation and cell death in fibrosis^39^. The *PPP1R13L* gene encodes a RELA-associated inhibitor that decreases p53/TP53 function and therefore suppresses the subsequent activation of apoptosis^40^. The *DUSP5* gene was also significantly downregulated in FFs, and methyl-CpG-binding protein 2 (MeCP2) increases the proliferation of cardiac fibroblasts and fibrosis by downregulating DUSP5^41^. In addition, we found that the *CD34* gene was significantly upregulated in NFs compared to FFs and human peripheral blood CD34+ cell transplantation can halt liver fibrosis and promote hepatic regeneration in chronic liver injury^42^.

In terms of growth factor binding, the *IGFBP5* and *FGFR3* genes were significantly downregulated in FFs compared to NFs. Insulin-like growth factor-binding protein 5 (IGFBP5) reduces liver fibrosis in chronic cholangiopathy^43^. The fibroblast growth factor 9 (FGF9) and 18 (FGF18) also inhibit myofibroblast differentiation in idiopathic pulmonary fibrosis and their biological effects are partially driven by FGFR3^44^. The CCN family of genes also represents matricellular proteins that modify signalling of other molecules, specifically those associated with the extracellular matrix. The *NOV* (*CCN3*) and *WISP2* (*CCN5*) genes were both significantly downregulated in FFs compared to NFs. NOV (CCN3) is a negative regulator of CTGF (CCN2) and a new endogenous inhibitor of the fibrotic pathway in an *in vitro* model of renal disease^45^. WISP2 (CCN5) is also a novel transcriptional regulator of the TGFβ signalling pathway^46^ and inhibits cardiac fibrosis^47^ as well as cell proliferation and motility in smooth muscle cells^48, 49^.

In addition, several extracellular matrix components were differentially expressed in FFs compared to NFs. The *PRG4* and *FBLN1* genes encode proteoglycan 4 and fibulin-1, respectively, and were significantly downregulated in FFs and in palmar fascia fibroblasts from patients with Dupuytren’s contracture^50^. Recombinant human PRG4 has anti-fibrotic effects and also decreases αSMA expression in lens epithelial cells activated with TGFβ2^51^. Scarless fetal wound healing is characterised by rapid and highly organised collagen deposition^27–29^ and we found a significant upregulation of the *COL6A6* gene in NFs. The *MMP-10*, *MMP-12* and *MMP-24* genes were also significantly downregulated in FFs. The decrease in the *MMP-10* gene could be explained by the fact that the *IL-6* gene was significantly upregulated in FFs and *IL-6* decreases *MMP-10* gene expression via the JAK2/STAT3 pathway^52^.

Fibrosis and cancer also share many cellular and molecular pathophysiological mechanisms, namely genetic and epigenetic changes, matrix contraction and remodelling, altered regulation of apoptosis, inflammation, and angiogenesis. It could in fact be argued that fibrosis and cancer represent a spectrum of the same disease and that fibrotic tissues have an increased risk of becoming cancerous compared to non-fibrotic tissues. Several oncogenes (*LMO3, MYB, BIRC3)* were significantly upregulated in FFs compared to NFs. The *LMO3* gene is a neuroblastoma-associated oncogene^53^ that encodes a LIM-domain-only protein involved in self-renewal, cell cycle regulation, and metastasis^54^. The *MYB* oncogene also plays a key role in cell proliferation and differentiation^55^ in leukaemia^56^, breast cancer^57^, and colon cancer^58^. The *BIRC3* gene is an inhibitor of apoptosis protein leading to apoptosis evasion^59^ and a predictor of aggressiveness and therapeutic resistance in glioblastoma^59^. Moreover, we found a significant downregulation of several tumour suppressor genes (*IGFBP5, WISP2, RASSF2)* in FFs compared to NFs. The *IGFBP5* gene is a tumour suppressor gene in melanoma and osteosarcoma^60^. *WISP*2 is a negative regulator of growth, migration, invasion, and a tumour suppressor in colorectal^61^, gastric^62^ and breast cancers^63^. The *RASSF2* gene is also a tumour suppressor gene in colorectal cancer^64^ and oral squamous cell carcinoma^65^.

We are setting up a fibrosis biobank of conjunctival tissues and fibroblast cell lines and we aim to validate our results in larger longitudinal studies in the future. A limitation of our study is that most of the patients in the fibrotic and non-fibrotic groups were on several anti-glaucoma eye drops and the effects of these medications on the gene expression profile remain largely unknown. This might in fact account for the degree of variability in gene expression noted among the NF cell lines. There were also both primary open angle and congenital glaucoma patients in the FF and NF groups, a lower number of females in the NF group, and the expression data were derived from primary fibroblast cell lines instead of the original conjunctival tissues. It is well established that Afro-Caribbean patients scar worse than white Caucasian patients. The majority of patients in our cohort were however white Caucasians and we found a large overlap of genes between the ‘all patients’ and ‘white Caucasians’ group comparisons.

In conclusion, we have identified a distinct fibrosis gene signature in the conjunctiva after glaucoma surgery. We have further mapped the differentially expressed genes to distinct pathways linked to smooth muscle contraction, inflammatory cytokines, immune mediators, extracellular matrix proteins, and oncogene expression. Several of the modules identified also contained potential drug targets that could be used to develop new anti-fibrotic treatments in the future. Fibrosis is a complex multifactorial disease and our genome-wide RNA-Seq study provides new insights into the mechanistic pathways driving the fibrotic process, as well as potential novel therapeutic targets and biomarkers of disease severity, in conjunctival fibrosis and other similar contractile scarring conditions in the eye and other tissues.

## Methods

### Patient Recruitment

We prospectively recruited glaucoma patients at the Moorfields Eye Hospital (London, UK) from September 2014 to September 2015, and collected conjunctival tissues at the time of glaucoma filtration surgery. All experimental protocols were approved by the London-Dulwich research ethics committee (REC reference 10/H0808/127) and the institutional approval committee at the University College London Institute of Ophthalmology. All the methods were carried out in accordance with the approved guidelines. All patients also gave written informed consent and the study adhered to the tenets of the Declaration of Helsinki. The inclusion criteria were: age (over 18 year-old) and patients planned to have glaucoma tube surgery. The exclusion criteria were previous conjunctival surgery other than glaucoma surgery. Patients were divided into 2 groups: patients who had previous glaucoma surgery and patients with no previous glaucoma surgery.

### Clinical Phenotype

We collected detailed clinical data on each patient, including age, ethnicity, gender, best-corrected visual acuity, intraocular pressures, cup-disc ratio, previous glaucoma surgeries, and anti-glaucoma medications. We also assessed each patient using the Moorfields bleb grading system. Central and maximal bleb areas and bleb height were graded on a scale of 1 to 5 (1 = 0%, 2 = 25%, 3 = 50%, 4 = 75%, 5 = 100%). Bleb vascularity was graded on a scale of 1 to 5 (1 = avascular, 2 = normal, 3 = mild hyperaemia, 4 = moderate hyperaemia, 5 = severe hyperaemia).

### Fibroblast Cell Lines

We established fibrotic fibroblast (FF) and non-fibrotic fibroblast (NF) primary cell lines from conjunctival tissues collected from patients with previous glaucoma surgery and patients with no previous glaucoma surgery, respectively. The conjunctival tissues were mechanically dispersed and the tissue fragments were placed in tissue culture dishes with Dulbecco’s modified Eagle’s medium (DMEM, Invitrogen), 10% fetal calf serum, 100 U/ml penicillin, 100 μg/ml streptomycin, and 2 mM L-glutamine at 37 °C with 5% CO_2_ as previously described^66^. Following outgrowth from the explant, the fibroblasts were trypsinised and cultured routinely in the above medium. Fibroblast cell lines in early passages 1–2 were used in the experiments.

### RNA Sequencing

The RNA was extracted from each fibroblast cell line using the RNeasy mini kit (Qiagen, UK) according to the manufacturer’s instructions. The RNA sequencing was performed at the UCL Genomics facility (London, UK) using TruSeq RNA Library Prep kit v2 (http://www.illumina.com/products/by-type/sequencing-kits/library-prep-kits/truseq-rna-v2.html). RNA quality was measured using Agilent 2100 Bioanalyzer (Agilent Technologies, Santa Clara, USA) and each sample was graded using an RNA integrity number equivalent (RINe) between 1 and 10. We used 500 ng of total RNA as input, based on quantification by the Agilent TapeStation RNA assay. Libraries were multiplexed into a single pool and sequenced across all four lanes of an Illumina NextSeq 500. Paired-end sequences of 43 nucleotides were generated after adapter removal.

### Differential Expression Analysis

The FASTQ files were assessed for quality control using FASTQC 0.11.2 (http://www.bioinformatics.bbsrc.ac.uk/projects/fastqc/). Adapter sequences were removed using Trimgalore (0.43) (https://www.bioinformatics.babraham.ac.uk/projects/trim_galore/). Paired-end sequence reads were aligned to the human GRCh38.p3 release 82 reference transcriptome and genome using STAR 2.4.2a^67^. Counts were summarised using HTSeq 0.6.1^68^ after read duplicates were marked using Picard MarkDuplicates (http://www.broadinstitute.github.io/picard) and subsequently removed using Samtools^69^. Differential expression analysis was performed between FF and NF sample groups based on summarised read counts using the DESeq2 (1.12.3) package^70^. Prior to modelling with DESeq2, read counts from the sex chromosomes were removed. DESeq2 utilises a negative binomial generalised linear model and normalised sample size through the packages median-of-ratios method. Read counts were fit to a model where condition is the factor of interest: FF and NF levels. Resulting *p* values were adjusted for multiple testing, as part of the DESeq2 analysis, using Benjamini-Hochberg correction^71^. Genes with more than a 2-fold change in expression and an adjusted *p* value < 0.05 were denoted as differentially expressed.

### Gene Ontology, KEGG, Disease association, Pathway commons, and WikiPathways Analyses

We used the list of differentially expressed genes to perform GO (gene ontology), KEGG (Kyoto Encyclopedia of Genes and Genomes), disease association, pathway commons, and WikiPathways analyses using WebGestalt^72^ and ClueGo^73^ with Cytoscape^74^ for visualisation.

### Protein Network Analysis

The set of genes that were differentially expressed (with an adjusted *p* value < 0.1) were used to build a sub-graph of the string network (https://string-db.org/). The sub-graph was converted into a weighted adjacency matrix, where the weights were derived from the STRING v10 confidence score (range 0 to 1000) of a functional association. Diagonal elements of the matrix, i.e. self-similarities, were set to a maximum confidence score of 1000. Functional association scores between proteins in the adjacency matrix were multiplied by -1 if the genes had opposite directions of expression fold change. A Euclidean distance matrix (between each column vector) was then generated between the columns of the adjacency matrix. A low value (distance) in the resulting distance matrix indicates that the two genes have similar interaction partners and direction of differential expression. We next applied agglomerative clustering to the distance matrix using the Ward metric. We assigned a high level function to each cluster by its most significant enrichment, using Fishers exact test enrichment (one of: extracellular matrix organisation, extracellular matrix, secreted, immune system process, signalling, immune response, response to stress, regulation of secretion by cell, immune system process, cell communication). The Function-Gene assignments were obtained from Ensembl-BioMart GO annotations, except for ‘secreted’ class, which was obtained from Ensembl-BioMart Signal-P predictions. Visual inspection suggested an appropriate number of clusters for further analysis as six, which gave a cophenetic distance of 0.69. The clusters were then imported into Cytoscape where the major connected components using the STRING interaction data from each cluster were displayed.

### Real-Time quantitative PCR

RT-qPCR reactions were performed using a Platinum quantitative PCR master mix (ThermoFisher Scientific, Hemel Hempstead, UK) on a CFX Real-Time PCR detection system (Bio-Rad, Hemel Hempstead, UK). The Taqman gene expression assays were: MYOCD (Hs00538071_m1), IL-6 (Hs00985639_m1), IL-33 (Hs00369211_m1), WISP2 (Hs00180242_m1), PRG4 (Hs00981633_m1), CD34 (Hs02576480_m1), RELB (Hs00232399_m1), IGFBP5 (Hs00181213_m1), MMP-10 (Hs00233987_m1), COL6A6 (Hs01029204_m1), LMO3 (Hs00998696_m1), and GAPDH (Hs02758991_g1) (ThermoFisher Scientific, Hemel Hempstead, UK). All mRNA values were normalised relative to that of GAPDH and triplicate experiments were performed for each gene. Statistical analysis was performed using the Student’s t-test to calculate statistically significant differences and individual *p* values. The validation of the RNA-Seq data with RT-qPCR was performed using the Spearman’s correlation of the mean values obtained from all samples normalised to either NF or FF samples. Statistically significant differences were expressed as *p < 0.05; **p < 0.01; ***p < 0.001.

## Electronic supplementary material

Supplementary Legends

S1

S2

S3

S4

S5

S6

S7

S8

S9
