# Supplementary material for: Genome-wide RNA-Sequencing analysis identifies a distinct fibrosis gene signature in the conjunctiva after glaucoma surgery
Source: Sci Rep. 2017 Jul 17;7:5644. doi: 10.1038/s41598-017-05780-5 (PMC5514109; doi:10.1038/s41598-017-05780-5)
Supplement: Supplementary file 1 — Supplementary Legends [file 41598_2017_5780_MOESM1_ESM.doc]

**Genome-wide RNA-Sequencing analysis identifies a distinct fibrosis gene signature in the conjunctiva after glaucoma surgery**

### Cynthia Yu-Wai-Man^1,2^ *, Nicholas Owen^2^, Jonathan Lees^3^, Aristides D. Tagalakis^4^, Stephen L. Hart^4^, Andrew R. Webster^1,2^, Christine A. Orengo^3^, Peng T. Khaw^1,2^

1. National Institute for Health Research (NIHR) Biomedical Research Centre at Moorfields Eye Hospital NHS Foundation Trust and UCL Institute of Ophthalmology, London, EC1V 9EL, United Kingdom
2. UCL Institute of Ophthalmology, London, EC1V 9EL, United Kingdom
3. Bioinformatics Research Group, UCL Institute of Structural and Molecular Biology, London, WC1E 6BT, United Kingdom
4. Experimental and Personalised Medicine Section, UCL Great Ormond Street Institute of Child Health, London, WC1N 1EH, United Kingdom

**Supplementary Legends**

**S1.** List of differentially expressed genes in FFs compared to NFs with more than a two-fold change and which are statistically significant (*p* < 0.05).

**S2.** List of shared and unique genes in the ‘all patients’ and ‘white Caucasians’ groups. The genes are differentially expressed with more than a two-fold change and *p* < 0.05.

**S3.** Directed acyclic graph (DAG) view of the Gene Ontology analysis of FFs compared to NFs. Red letters indicate enriched ontology groups, number of genes in each group, and adjusted p (adjP) values.

**S4.** GO (Gene Ontology) enrichment analysis between FFs and NFs. For each GO category, the first row lists its sub-root (biological process, molecular function or cellular component), category name, and corresponding GO ID. The second row lists number of reference genes in the category (C), number of genes in the gene set and also in the category (O), expected number in the category (E), ratio of enrichment (R), *p* value from hypergeometric test (rawP), and *p* value adjusted by the multiple test adjustment (padj). For each gene, the table lists the user uploaded ID and value, Entrez Gene ID, Ensembl Gene Stable ID, Gene symbol, and description.

**S5.** KEGG (Kyoto Encyclopedia of Genes and Genomes) analysis between FFs and NFs. For each KEGG pathway, the first row lists the KEGG pathway name and corresponding KEGG ID. The second row lists number of reference genes in the category (C), number of genes in the gene set and also in the category (O), expected number in the category (E), ratio of enrichment (R), *p* value from hypergeometric test (rawP), and *p* value adjusted by the multiple test adjustment (padj). For each gene, the table lists the user uploaded ID and value, Entrez Gene ID, Ensembl Gene Stable ID, Gene symbol, and description.

**S6.** Disease association analysis between FFs and NFs. For each gene set, the first row lists the gene set name and corresponding Gene Set ID. The second row lists number of reference genes in the category (C), number of genes in the gene set and also in the category (O), expected number in the category (E), ratio of enrichment (R), *p* value from hypergeometric test (rawP), and *p* value adjusted by the multiple test adjustment (padj). For each gene, the table lists the user uploaded ID and value, Entrez Gene ID, Ensembl Gene Stable ID, Gene symbol, and description.

**S7.** Pathway commons analysis between FFs and NFs. For each gene set, the first row lists the gene set name and corresponding Gene Set ID. The second row lists number of reference genes in the category (C), number of genes in the gene set and also in the category (O), expected number in the category (E), Ratio of enrichment (R), *p* value from hypergeometric test (rawP), and *p* value adjusted by the multiple test adjustment (padj). For each gene, the table lists the user uploaded ID and value, Entrez Gene ID, Ensembl Gene Stable ID, Gene symbol, and description.

**S8.** WikiPathways analysis between FFs and NFs. For each gene set, the first row lists the gene set name and corresponding Gene Set ID. The second row lists number of reference genes in the category (C), number of genes in the gene set and also in the category (O), expected number in the category (E), ratio of enrichment (R), *p* value from hypergeometric test (rawP), and *p* value adjusted by the multiple test adjustment (padj). For each gene, the table lists the user uploaded ID and value, Entrez Gene ID, Ensembl Gene Stable ID, Gene symbol, and description.

**S9.** Protein network analysis between FFs and NFs. List of differentially expressed genes in each of the 6 clusters with Ensembl-ID, fold change of FFs compared to NFs, and padj values.
