# Supplementary figures and images for: Genome-wide RNA-Sequencing analysis identifies a distinct fibrosis gene signature in the conjunctiva after glaucoma surgery
Source: Sci Rep. 2017 Jul 17;7:5644. doi: 10.1038/s41598-017-05780-5 (PMC5514109; doi:10.1038/s41598-017-05780-5)

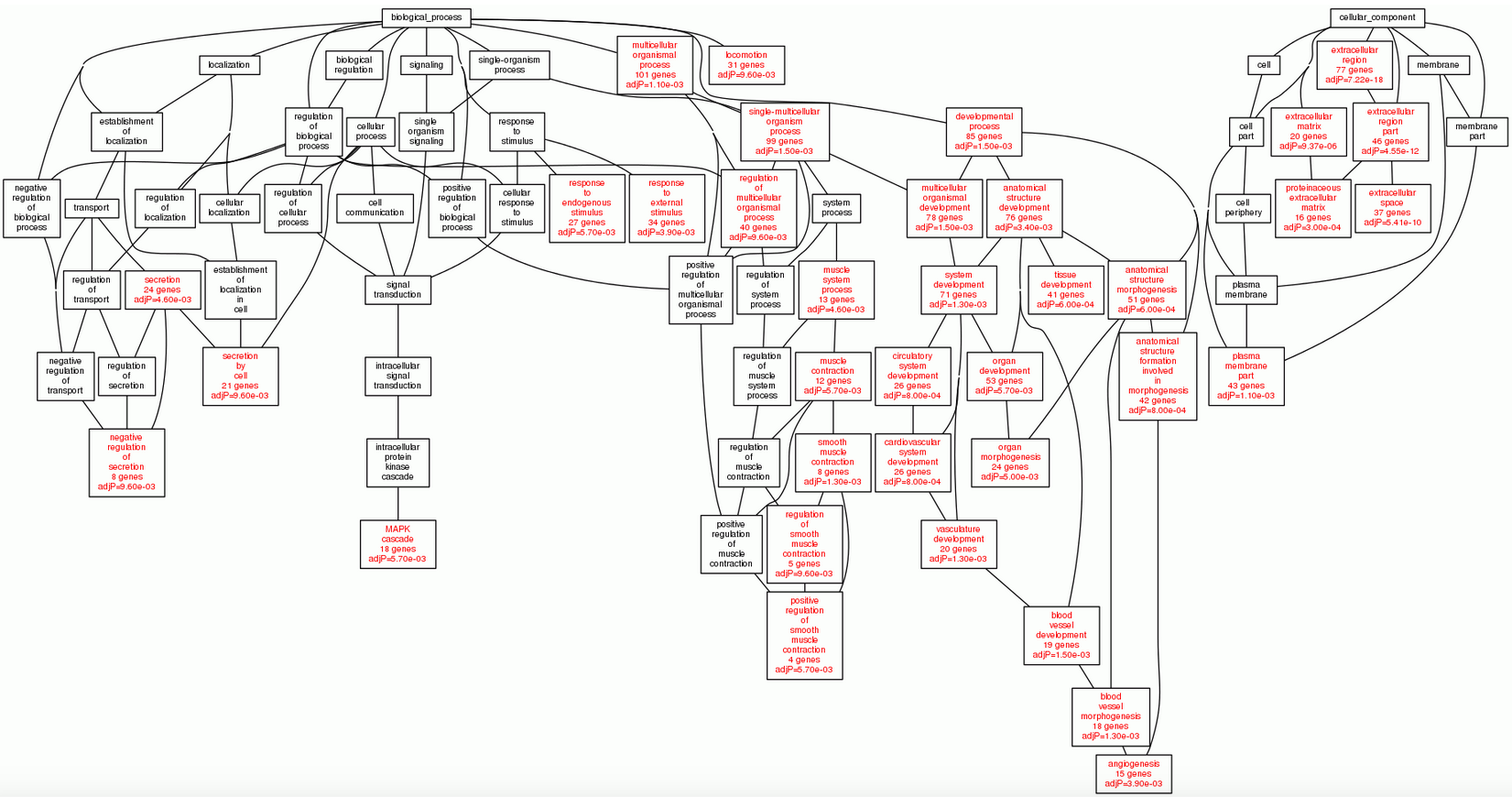

Supplement: Supplementary file 4 — S3 [file 41598_2017_5780_MOESM4_ESM.doc]
